# Natural History of Swallow Function during the Three-Month Period after Stroke

**DOI:** 10.3390/geriatrics4030042

**Published:** 2019-07-09

**Authors:** Viridiana Arreola, Natàlia Vilardell, Omar Ortega, Laia Rofes, Desiree Muriana, Ernest Palomeras, Daniel Álvarez-Berdugo, Pere Clavé

**Affiliations:** 1 Gastrointestinal Physiology Laboratory, Department of Surgery, Hospital de Mataró, Universitat Autònoma de Barcelona, 08304 Mataró, Spain; 2 Centro de Investigación Biomédica en Red de enfermedades hepáticas y digestivas (CIBERehd), Instituto de Salud Carlos III, 08036 Barcelona, Spain; 3 Neurology Unit, Hospital de Mataró, Universitat Autònoma de Barcelona, 08304 Mataró, Spain; 4 Fundació Institut d’Investigació en Ciències de la Salut, Germans Trias i Pujol, 08916 Badalona, Spain

**Keywords:** deglutition, deglutition disorders, function recovery, neurophysiology, stroke

## Abstract

Oropharyngeal dysphagia is a prevalent complication following stroke (PS-OD), and one that is sometimes spontaneously recovered. This study describes the natural history of PS-OD between admission and three months post-stroke, and the factors associated with its prevalence and development. PS-OD was assessed with the volume-viscosity swallow test (V-VST) in all stroke patients on admission and at the three-month follow-up. We analyzed clinical, demographic, and neuroanatomical factors of 247 older post-stroke patients (National Institute of Health Stroke Scale (NIHSS) = 3.5 ± 3.8), comparing among those with PS-OD the ones with and without spontaneous recovery. PS-OD prevalence on admission was 39.7% (34.0% impaired safety; 30.8%, efficacy) and 41.7% (19.4% impaired safety; 39.3%, efficacy) at three months. Spontaneous swallow recovery occurred in 42.4% of patients with unsafe and in 29.9% with ineffective swallow, associated with younger age and optimal functional status. However, 26% of post-stroke patients developed new signs/symptoms of ineffective swallow related to poor functional, nutritional and health status, and institutionalization. PS-OD prevalence on admission and at the three-month follow-up was very high in the study population. PS-OD is a dynamic condition with some spontaneous recovery in patients with optimal functional status, but also new signs/symptoms can appear due to poor functionality. Regular PS-OD monitoring is needed to identify patients at risk of nutritional and respiratory complications.

## 1. Introduction

Stroke is one of the most prevalent causes of death in Europe and the most important cause of morbidity and disability, and its presence significantly increases healthcare utilization and costs [1]. Post-stroke oropharyngeal dysphagia (PS-OD) is an important and frequent complication identified in up to 45% of patients by means of the volume-viscosity swallowing test (V-VST), remaining in 66% of discharged patients [2]. PS-OD patients are characterized by delayed time to laryngeal vestibule closure (LVC) and tongue weakness with low bolus propulsion force that lead to nutritional and respiratory complications, such as malnutrition and aspiration pneumonia [3]. In a recent publication, PS-OD was found to be a prognostic factor for respiratory infections at three and 12 months after discharge [2].

Despite the high percentage of PS-OD in the acute phase, spontaneous swallowing recovery may occur in about half of the patients in the weeks following the stroke episode [4]. This recovery is mainly related to cortical reorganization (neuroplasticity) and an increase in pharyngeal motor representation in the contralesional motor cortex [5,6,7]. On the other hand, patients who do not recover from PS-OD remain with severe impaired biomechanics of the swallow response; prevalent impairments include disrupted integration of pharyngeal sensory inputs and reduced cortical excitability of the efferent pathways without physiologic hemispheric dominance (lack of increased motor representation in the contralesional motor cortex) [8]. Thus, these patients continue to have swallowing dysfunctions with associated complications that include malnutrition, dehydration, and aspiration pneumonia, with high mortality rates and poor quality of life [2]. 

Clinical and neuroanatomical stroke factors associated with PS-OD and unsafe swallow in acute post-stroke patients have been widely studied: Age, stroke severity, previous stroke event, and a larger volume of stroke lesion are the most common reported risk factors associated with PS-OD [2,9,10]. In addition, PS-OD is an independent risk factor for prolonged hospital stay, institutionalization after discharge, poorer functional capacity, and increased mortality three months after stroke [2]. However, factors associated with spontaneous recovery from PS-OD, as well as the evolution of the swallowing impairments that these patients present from discharge to the chronic phase, have not yet been studied. There are several clinical tests to monitor this evolution; the V-VST is a clinical test to assess clinical signs of impaired efficacy and safety of swallow that can be done at the bedside and that has good psychometric properties [11], allowing to evaluate the changes between discharge and the recovery period. Impaired efficacy of swallow is related to the development of nutritional complications and is characterized by the presence of impaired labial seal, oral residue, pharyngeal residue, and piecemeal deglutition. On the other hand, impaired safety of swallow is related to the development of respiratory complications and is characterized by the presence of penetrations and aspirations, translated into cough, wet voice, and oxygen desaturation (≥3 points) during the V-VST [11].

The natural history of PS-OD and its recovery is not fully understood and although there are many studies in the acute and chronic stroke phase, little is known about the in between period (admission to three months from stroke onset). The aim of this study was to assess the natural history of PS-OD from admission to the chronic phase at the three-month follow-up. We aimed to describe the prevalence of patients with spontaneous clinical recovery of the swallowing function and to determine those demographic, clinical and neuroanatomical stroke factors associated with changes in the swallowing function during the chronic post-stroke stage.

## 2. Material and Methods

### 2.1. Study Design and Population

This study was an observational, prospective, longitudinal study of patients without previous swallowing dysfunction, consecutively admitted on confirmed stroke diagnosis to a general hospital (May 2012 to September 2014). Swallowing function was assessed by V-VST during the first 48 h after admission to the neurology unit and reevaluated three months later. Only patients that were alive three months post-stroke were included in the study. None of the patients received any active intervention for PS-OD within the study period. Patients received standard clinical practice that was based on fluid adaptation (volume and viscosity) according to the results of the V-VST, and textural adaptation according to their needs (supervised by the Hospital’s dietitian). The study protocol was approved by the ethical committee of the hospital (protocol code 17/11), and was conducted according to the principles and rules laid down in the Declaration of Helsinki and its subsequent amendments. 

### 2.2. Data Collected

Sociodemographic and clinical data were collected on admission and at the three-month follow-up visit. During hospital stay, stroke etiology, location (side and territory affected), and volume of the stroke lesion were collected from medical and neuroimaging techniques (computed tomography scan and magnetic resonance imaging) source reports. Stroke severity was scored according to the National Institute of Health Stroke Scale (NIHSS) that quantifies stroke severity based on weighted evaluation findings [12]. Stroke type and subtype were classified according to the Oxford Community Stroke Project that divides cerebral infarction into total anterior circulation (TACI), partial anterior circulation (PACI), posterior circulation (POCI), and lacunar (LACI) syndromes [13]. The length of hospital stay in the neurology unit was registered. Functional capacity was assessed using the Barthel Index [14] that evaluates the capacity of the patient to perform the activities of daily living (functional independence), and the modified Rankin Scale that measures the degree of disability or dependence in the daily activities of people who have suffered a stroke [15]. Nutritional status was evaluated according to the Mini Nutritional Assessment short form (MNA-sf), a brief nutritional questionnaire with a score from 0 to 14 that classifies patients into well-nourished (12–14), at risk of malnutrition (11–8), and malnourished (7–0) [16]; it was performed on admission and at the follow-up visit. Patient destinations on discharge and place of residence three months later were registered. Patient quality of life was assessed with the European Quality of Life Scale (EQ-5D-3L) questionnaire that takes into account the following five dimensions: Mobility, self-care, usual activities, pain/discomfort, and anxiety/depression [17]; it was evaluated at the three-month follow-up visit. Clinical questionnaires/scales were acquired by a clinician (neurologist); nutritional evaluation was performed by the study dietitian.

### 2.3. Clinical Assessment 

Swallowing function was assessed with the validated V-VST within the first 48 h after stroke in the neurology unit, and reevaluated three months later in the gastrointestinal physiology laboratory also using the V-VST. All the clinicians evaluating the swallowing function were trained personnel from the dysphagia team at Hospital de Mataró and applied the same protocol algorithm [11,18]. The V-VST uses different bolus volumes (5, 10, and 20 mL) and the following viscosities: 250 mPa·s (called nectar by the National Dysphagia Diet (NDD) nomenclature), <50 mPa·s (called thin liquid by the NDD), and 3500 mPa·s (called spoon-thick by the NDD). These viscosities were obtained by using mineral water for thin liquid and the modified starch thickener (Resource ThickenUp^®^, Nestlé Health Science) according to our previous studies [11]. V-VST allows the safety and efficacy of swallowing of each bolus to be evaluated with minimum risk to the patient. Impaired efficacy of swallow is identified by the presence of any of the following clinical signs during the test: Oral residue, impaired labial seal, fractional swallow, or pharyngeal residue. Impaired safety of swallow is defined by: The presence of wet voice, cough, and decrease in oxygen saturation ≥3% from the basal level [18]. The test begins with 250 mPa·s at low volume and continues with 10 and 20 mL if no safety impairments occur. Then, it continues with the same pattern with liquid and finishes with 3500 mPa·s. On the other hand, if impaired safety occurs in any volume or viscosity, the explorer continues the V-VST with 3500 mPa·s at low volume. Finally, if there is any safety impairment with 3500mPa·s, the test is stopped. The V-VST is a clinical screening tool with high sensitivity and specificity in the assessment of dysphagia (94% and 88%) and in detecting impaired efficacy of swallow (79% and 75%) [19]. 

### 2.4. Data Analysis and Statistical Methods 

Quantitative parameters were described as mean ± standard deviation (SD) and comparisons were assessed by the non-parametric Kruskal–Wallis and Mann–Whitney tests. Discrete variables were expressed as median (Interquartile range). Qualitative parameters were described by relative and absolute frequencies and compared by the Fischer’s exact test. Odds ratio and 95% confidence interval (CI) were provided and *p*-values <0.05 were considered statistically significant. Statistical analyses were performed with the GraphPad Prism 6 software (GraphPad Software, San Diego, CA, USA).

The results of the V-VST (prevalence of impaired efficacy and safety of swallow) performed at the three-month follow-up visit were compared with the results on admission. Demographical, clinical, and neuroanatomical characteristics of those patients with changes in swallowing function (recovery or worsening) were compared with those from post-stroke patients without changes.

## 3. Results

### 3.1. Study Population

We assessed 247 post-stroke patients (72.3 ± 11.9 years, 59.5% male) using the V-VST on admission and, in the chronic phase, at 3-month follow up (139.3 ± 51.9 days from stroke). Post-stroke patients presented mild ischemic stroke (NIHSS 3.5 ± 3.8), mostly affecting the left side and the partial anterior circulation (PACI) leading to mild functional disability on admission, slightly improved at the follow-up visit with high incidence of self-reported symptoms of pain/discomfort and anxiety/depression (Table 1).

### 3.2. Prevalence of OD: Admission and the 3-Month Follow-Up

Prevalence of PS-OD on admission was 39.7% (34.0% of patients with impaired safety of swallow and 30.8% with impaired efficacy). Prevalence of PS-OD three months after stroke was 41.4% (19.1% with impaired safety of swallow and 39.0% with impaired efficacy). 

**Figure 1:**
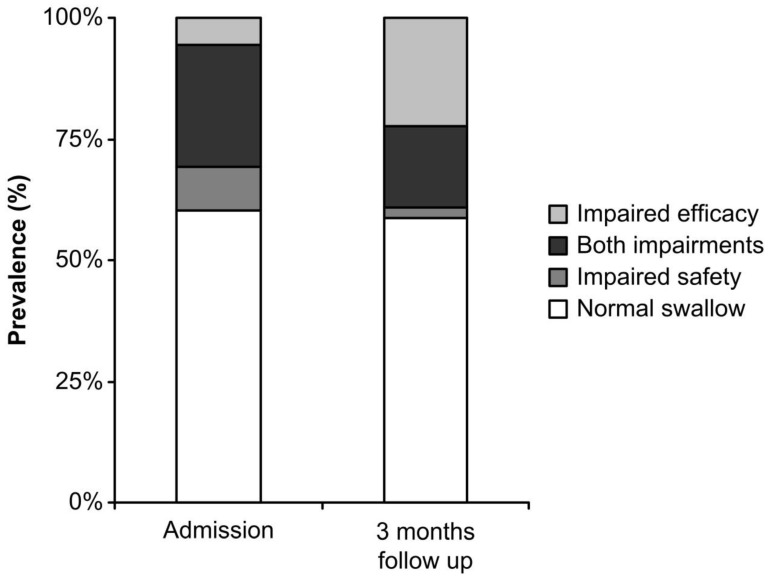
Prevalence of normal swallow and prevalence of clinical signs of impaired safety and efficacy of swallow on admission and at the 3-month follow-up.

On admission, impaired safety of swallow was detected in 85 post-stroke patients, 42.4% (36) of whom spontaneously recovered swallowing function, and 57.6% (49) continued to have safety impairments at the 3-month follow-up visit (Figure 1).

**Figure 2:**
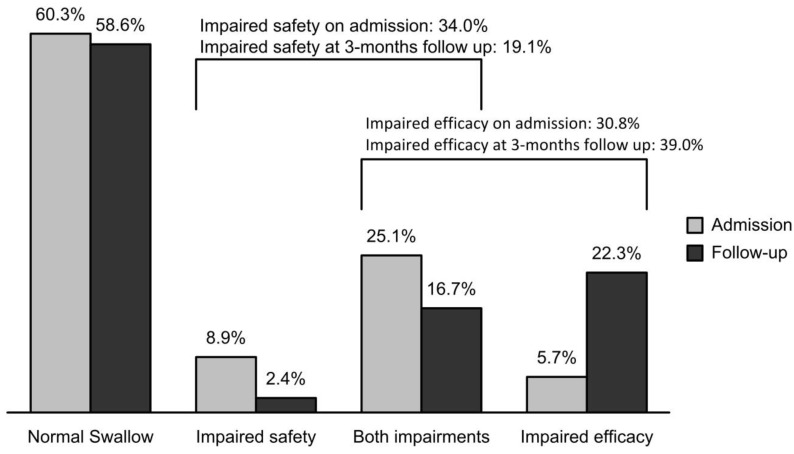
Description of development of clinical signs of oropharyngeal dysphagia on admission and at the 3-month follow up.

Impaired efficacy of swallow was detected in 77 post-stroke patients on admission, 29.9% (23) of whom recovered and 70.1% (54) continued to have impairments at the 3-month follow-up visit. Moreover, at the follow-up visit, clinical signs of impaired efficacy of swallow appeared in up to 25.9% (44) of post-stroke patients who had had no previous clinical signs (Figure 2). 

### 3.3. Factors Associated with the Recovery of Impaired Safety of Swallow 

Spontaneous recovery of safety of swallow was associated with age, no previous heart diseases, and nearly optimal functional capacity (Barthel Index ≥90) at the follow-up visit. There was a trend of recovery of safety of swallow in those post-stroke patients with middle cerebral artery (MCA) infarction (Table 2).

### 3.4. Factors Associated with the Recovery of Efficacy of Swallow 

Spontaneous recovery of efficacy of swallow was linked to absence of previous heart diseases. Moreover, post-stroke patients with chronic efficacy impairments had significantly higher prevalence of anxiety/depression compared with post-stroke patients who recovered (Table 3).

### 3.5. Factors Associated with the Worsening of Swallowing Function During Follow up

The appearance de novo of impaired efficacy was associated with poorer pre-stroke functional status, infarction of vascular territories other than posterior circulation (POCI), institutionalization on hospital discharge, and poorer functional status. Post-stroke patients with new impaired efficacy of swallow presented a significantly poorer nutritional status and lower perception of health status with a higher degree of pain/discomfort, difficulties in mobility, and self-care dependency compared with post-stroke patients without signs of impaired efficacy at follow up (Table 4). We did not find any patients presenting de novo signs of impaired safety of swallow during follow up.

## 4. Discussion

The main result of this study is that PS-OD is a dynamic condition with some spontaneous recovery during the chronic post-stroke stage, but also with the appearance of new impairments in swallowing function in some vulnerable patients who did not have PS-OD on admission. Our results suggest that systematic and regular PS-OD monitoring is needed in this phase to identify those post-stroke patients with high risk of nutritional and respiratory complications. 

Prevalence of PS-OD in our mild-severity stroke population was very high in the acute phase (39.7%). Almost 42% of the post-stroke patients with clinical signs of unsafe swallow and almost 30% with clinical signs of impaired efficacy of swallow on admission spontaneously recovered their impairments in the chronic phase. However, PS-OD prevalence remained similar at both time points due to the appearance of new signs of impaired safety of swallow. At the follow-up assessment, we detected 44 new clinical diagnoses of impaired efficacy of swallow in PS-OD patients. Moreover, we identified clinical and neurotopographical stroke factors significantly associated with recovery and/or deterioration of the swallowing function in the post-stroke population.

PS-OD prevalence in this study is similar to that reported in other studies where PS-OD was screened using multiple variations of the water clinical test in the acute stroke phase [20,21,22,23,24]. We used the V-VST, a clinical test for screening swallowing dysfunction, recently validated in different phenotypes of patients [11,19] including post-stroke populations, and that shows high sensitivity and specificity for impaired safety of swallow (84.2% and 64.3%, respectively) and aspirations (88.2% and 71.4%, respectively) [25]. In addition, the V-VST evaluates efficacy impairments, giving a more comprehensive view of the patient’s swallowing function than water swallow tests. 

The V-VST allowed us to detect an unexpectedly high prevalence of PS-OD in the chronic stage. This was due to prevalence of impaired efficacy of swallow, while prevalence of unsafe swallowing was reduced by more than 40% at the three-month follow-up. This improvement in the safety of swallow is similar to previous reports which described incidence of improvements of swallowing in over 50% of post-stroke patients within the first week post-stroke [22,26,27]. We also found that spontaneous recovery from unsafe swallowing was significantly associated with younger age, lack of previous heart diseases, and optimal functional status at three months. There are few studies that have evaluated the factors significantly associated with the recovery of swallowing impairments after stroke, but they confirm our observations and associated improvements in nutritional intake with functional improvements and younger age [28,29]. Other authors associated chronic dysphagia with low functional capacity (Barthel Index), impaired consciousness, motor impairments, and larger lesions affecting the frontal and insular cortex [27,29,30,31]. Regarding the relationship between the affected territory and the recovery of the swallowing function, we found that patients with MCA affection were more frequent (nearly statistically significant) in those post-stroke patients who recovered from impaired swallow, probably related to the fact that patients with supratentorial infarcts recovered swallowing function more than patients with infratentorial infarcts. 

We also found that nearly 30% of post-stroke patients who recovered efficacy of swallow in the chronic phase were significantly associated with not having previous heart disease, probably due to the fact that those patients were younger than the ones with a previous heart disease. In addition, patients who did not recover the efficacy of swallow presented higher anxiety and depression symptoms. No data related to the improvement of efficacy impairments have been previously described because available published studies assessed dysphagia by means of modified water tests that do not measure specific signs and symptoms of impaired efficacy of swallow. 

One of the most relevant results of our study was that 26% of post-stroke patients showed new signs or symptoms of impaired efficacy of swallow at the follow-up visit. The reasons for that increase are multiple, as many of these PS-OD patients are older and oropharyngeal dysphagia has been published as a major geriatric syndrome—meaning that dysphagia could have multiple causes [32]. PS-OD patients are more fragile, have more functional impairment or deterioration, institutionalization, and malnutrition, and all this can lead to dysphagia and impaired efficacy of swallow. Although these new signs of impaired efficacy are not directly caused by stroke, they form part of the natural history of stroke. A recent paper found that, apart from specific characteristics determined by the acute stroke, premorbid conditions such as sarcopenia also have an impact on the pathophysiology of PS-OD [33]. In the chronic phase, other authors have reported on the inability to return to pre-stroke swallowing capacity (13%), with reduced swallowing function in 50% of patients [26]. In our study, factors associated with the appearance of de novo were institutionalization on hospital discharge, suboptimal functional capacity, poor nutritional status, and low self-reported quality of life with pain or discomfort and difficulties in mobility and self-care tasks at follow up. Low self-reported quality of life using the European Quality of Life questionnaire (EuroQoL) was previously reported in a cohort of untreated head and neck cancer patients with oropharyngeal dysphagia, who reported higher incidence of functional, physical, and emotional problems [34]. Factors associated with the appearance of inefficacy of swallowing have not been described, but a previous one-year follow-up study also noticed a major increase and variation in the prevalence of oral cavity residue (clinical signs of impaired efficacy of swallow) in the clinical evaluation of dysphagia from baseline (5%) to one month after stroke (50%) [35]. In addition, the association between chronic dysphagia and poor outcome (malnutrition, sarcopenia) is well-known [36]. 

A possible limitation of our study is that the V-VST was performed by different clinicians at each time point. However, we emphasize that all the clinicians were trained and experienced in dysphagia assessment and they used the same algorithm and protocol. Moreover, the V-VST presents good inter-rater agreement (Kappa coefficient of 0.628 (95% CI = 0.45–0.78)) when performed by well-trained professionals [19]. Another possible limitation is that patients from this study come from a bigger cohort (31.1% mortality in those with OD) and only those patients who were alive three- months post-stroke were included in the paper, in order to describe the prevalence of spontaneous clinical recovery of the swallowing function. Thus, this patient selection should be taken into account when interpreting the data.

In summary, we found that while many post-stroke patients recovered their ability to swallow safely during the first three months after stroke, a large proportion of patients continued to have safety and efficacy dysfunctions, and also many presented new clinical signs of ineffective swallow. Thus, it is important to assess swallowing function not only in the acute phase, but also in the chronic post-stroke phase in order to detect changes in swallowing and to establish the appropriate interventions to prevent poor outcome [37]. In addition, as treatment of PS-OD is changing from compensatory strategies to the enhancement of brain plasticity, both to recover swallow function and to improve brain-related swallowing dysfunction [38], our results suggests that randomized control studies assessing these new techniques should include control groups monitoring the spontaneous development of swallowing function. 

## Figures and Tables

**Table 1:** Demographic, clinical, and nutritional characteristics of post-stroke patients on admission and at 3-month follow up.

**Sample**	247
**Age (years)**	72.3 ± 11.9
**Sex (male)**	59.5% (147)
**Previous heart disease**	25.5% (63)
**NIHSS on admission (mean ± SD)** **Score ≤6 points (%)**	2(1–4) 87.0% (215)
**Type of Stroke**	
Ischemic Hemorrhagic Cerebral venous thrombosis	95.5% (236) 4.1% (10) 0.4% (1)
**Stroke Lateralization**	
Left hemisphere Right hemisphere Bilateral Brain stem Not specified	44.1% (109) 29.6% (73) 1.2% (3) 6.9% (17) 18.2% (45)
**Territory Infarction**	
MCA PCA AChA Watershed Basilar Vertebral	64.3% (117) 14.8% (27) 8.2% (15) 2.2% (4) 8.8% (16) 1.6% (3)
**Lesion Location**	
Supratentorial Infratentorial	89.6% (163) 10.4% (19)
**Stroke Diagnosis**	
PACI TACI POCI LACI Not specified	41.3% (102) 6.1% (15) 15.8% (39) 32.4% (80) 4.4% (11)
**Volume of stroke lesion (cc)**	11.8 ± 28.5
**Hospital length of stay (days)**	6.5 ± 3.5
**Institutionalization on discharge**	21.4% (52)
**Barthel Index**	
Pre-stroke event On discharge At follow-up visit	100 (100–100) 100 (50–100) 100 (80–100)
**Rankin Scale**	
Pre-stroke event On discharge At follow-up visit	0 (0–1) 2 (0–3) 2 (0–2)
**MNA-sf at follow-up visit (mean ± SD)** **≤11points**	13 (12–13) 30% (70)
**EQ-5D-3L at Follow-Up Visit**	
Mobility dysfunctions (%) Self-care dependency (%) Dependency in daily life activities (%) Pain/discomfort (%) Anxiety/depression symptoms (%) Perception of healthy status (mean ± SD)	26.7% (63) 23.7% (56) 26.3% (62) 41.5% (98) 52.1% (123) 68.2 ± 19.2

NIHSS: National Institute of Health Stroke Scale; MCA: Middle cerebral artery; PCA: Posterior cerebral artery; AChA: Anterior choroidal artery; PACI: Partial anterior circulation infarct; LACI: Lacunar infarct; POCI: Posterior circulation infarct; TACI: Total anterior circulation infarct; EQ-5D-3L: European Quality of Life Scale.

**Table 2:** Factors associated with recovered safety of swallow.

Factors	Recovery of Impaired Safety of Swallow at 3 Months	Maintained Impaired Safety of Swallow at 3 Months	OR (CI 95%); *p*-Value
**Sample (n)**	36	49	-
**Age (mean ± SD)**	73.1 ± 11.2	78.6 ± 9.4	0.009
**Sex (male) (%)**	61.1	53.1	1.4 (0.6–3.3); 0.512
**No previous heart disease (%)**	86.1	61.2	3.9 (1.3–11.9); 0.015
**Pre-stroke Rankin score (median (IQ range))**	0 (0–0)	0 (0–1)	0.061
**NIHSS on admission (median (IQ range))**	4 (2–5)	4 (1–7)	0.890
Score ≤6 points (%)	83.3	75.5	2.2 (0.7–7.0); 0.188
**Stroke lateralization**			
Left hemisphere (%) Right hemisphere (%)	62.1 37.9	65.0 32.5	0.8 (0.3–2.4); 0.806 1.3 (0.5–3.5); 0.798
**Territory infarction**			
MCA infarction (%)	81.5	52.5	3.4 (1.0–10.8); 0.058
**Lesion location**			
Supratentorial (%) Infratentorial (%)	88.9 11.1	81.1 18.9	1.9 (0.4–8.0); 0.498
**Stroke diagnosis**			
PACI (%) TACI (%) POCI (%) LACI (%)	54.5 15.2 12.1 18.2	37.0 17.4 21.7 23.9	2.1 (0.8–5.1); 0.169 0.8 (0.3–2.9); 1.000 0.5 (0.1–1.7); 0.374 0.7 (0.2–2.2); 0.591
**Volume of stroke lesion (cc) (mean ± SD)**	21.2 ± 40.8	13.1 ± 23.0	0.305
**Institutionalization on discharge (%)**	30.6	40.2	0.6 (0.3–1.6); 0.490
**Barthel Index on discharge (median (IQ range))**	70 (40–100)	80 (40–100)	0.864
**Barthel Index at follow-up visit (median (IQ range))**	100 (80–100)	70 (70–100)	0.057
Barthel ≥90 points (%)	60.0	35.4	2.9 (1.2–7.3); 0.039
**MNA-sf at follow-up visit (median (IQ range))**	11 (11–14)	12 (11–14)	0.668
**EQ-5D-3L:**			
Mobility dysfunctions (%)	39.4	52.4	0.6 (0.2–1.5); 0.352
Self-care dependency (%)	42.4	40.5	1.1 (0.4–2.7); 1.000
Dependency in daily life activities (%)	36.3	52.4	0.5 (0.2–1.3); 0.243
Pain/discomfort (%)	54.5	52.4	1.1 (0.4–2.7); 1.000
Anxiety/depression symptoms (%)	51.5	64.3	0.6 (0.2–1.5); 0.346
Perception of healthy status (mean ± SD)	68.0 ± 19.7	67.4 ± 19.1	0.886

NIHSS: National Institute of Health Stroke Scale; MCA: Middle cerebral artery; PACI: Partial anterior circulation infarct; LACI: Lacunar infarct; POCI: Posterior circulation infarct; TACI: Total anterior circulation infarct; EQ-5D-3L: European Quality of Life Scale; IQ: Interquartile.

**Table 3:** Factors associated with recovered efficacy of swallow.

Factors	Recovery of Efficacy of Swallow at 3 Months	Maintained Impaired Efficacy of Swallow at 3 Months	OR (CI 95%); *p*-Value
**Sample (n)**	23	54	-
**Age (mean ± SD)**	75.1 ± 8.5	77.6 ± 10.1	0.106
**Sex (male) (%)**	60.9	55.5	1.2 (0.5–3.4); 0.803
**No previous heart disease (%)**	87.0	59.3	4.6 (1.2–17.3); 0.019
**Pre-stroke Rankin score (median (IQ range))**	0 (0–2)	0 (0–1)	0.947
**NIHSS on admission (median (IQ range))** Score ≤6 points (%)	2 (1–5) 86.9	3 (1–11) 74.1	0.205 3.3 (0.7–16.2); 0.207
**Stroke lateralization**			
Left hemisphere (%) Right hemisphere (%)	60.0 40.0	61.3 36.4	0.9 (0.3–2.7); 1.000 1.2 (0.4–3.7); 0.788
**Territory infarction**			
MCA infarction (%)	75.0	59.1	1.7 (0.5–5.7); 0.402
**Lesion location**			
Supratentorial (%) Infratentorial (%)	95.0 5.0	85.4 14.6	3.3 (0.4–29.1); 0.410
**Stroke diagnosis**			
PACI (%) TACI (%) POCI (%) LACI (%)	52.2 4.3 26.1 17.4	41.2 15.7 23.5 19.6	1.6 (0.6–4.2); 0.452 0.3 (0.0–2.3); 0.267 1.3 (0.4–4.1); 0.766 0.9 (0.3–3.5); 1.000
**Volume of stroke lesion (cc) (mean ± SD)**	26.0 ± 48.7	18.7 ± 36.2	0.990
**Institutionalization on discharge (%)**	21.7	40.7	0.126
**Barthel Index on discharge (median (IQ range))**	90 (70-100)	80 (50-90)	0.441
**Barthel Index at follow-up visit (median (IQ range))** Barthel ≥90 points (%)	90 (80–100) 54.5	90 (50–100) 43.3	0.210 1.6 (0.6–4.3); 0.450
**MNA-sf at follow-up visit (median (IQ range))**	12 (10–14)	12 (11–14)	0.928
**EQ-5D-3L:**			
Mobility dysfunctions (%)	45.5	46.0	0.9 (0.4–2.7); 1.000
Self-care dependency (%)	45.5	36.0	1.5 (0.5–4.1); 0.600
Dependency in daily life activities (%)	45.5	48.0	0.9 (0.3–2.5); 1.000
Pain/discomfort (%)	50.0	46.0	1.2 (0.4–3.2); 0.802
Anxiety/depression symptoms (%)	36.4	66.0	3.6 (1.3–10.3); 0.021
Perception of healthy status (mean ± SD)	63.8 ± 16.4	66.9 ± 19.3	0.523

NIHSS: National Institute of Health Stroke Scale; MCA: Middle cerebral artery; PACI: Partial anterior circulation infarct; LACI: Lacunar infarct; POCI: Posterior circulation infarct; TACI: Total anterior circulation infarct; EQ-5D-3L: European Quality of Life Scale; IQ: Interquartile.

**Table 4:** Factors significantly associated with worsened efficacy of swallowing at 3 months post-stroke.

Factors	No Impaired Efficacy of Swallow at 3 Months	New Diagnosis of Impaired Efficacy of Swallow at 3 Months	OR (CI 95%); *p*-Value
**Sample (n)**	126	44	-
**Age (mean ± SD)**	69.3 ± 12.8	72.8 ± 10.7	0.106
**Sex (male) (%)**	65.1	47.7	2.0 (1.0–4.1); 0.050
**No previous heart disease (%)**	78.6	75.0	0.8 (0.4–1.8); 0.676
**Pre-stroke Rankin score (median (IQ range))**	0 (0–0)	0 (0–0)	0.045
**NIHSS on admission (median (IQ range))** Score ≤6 points (%)	2 (1–3) 86.5	2 (1–3) 93.2	0.544 1.2 (0.3–4.9); 0.720
**Stroke lateralization**			
Left hemisphere (%) Right hemisphere (%)	65.0 32.5	62.1 37.9	0.9 (0.4–2.0); 1.000 1.0 (0.5–2.2); 1.000
**Territory infarction**			
MCA infarction	49.2	33.3	1.9 (0.9–4.0); 0.104
**Lesion location**			
Supratentorial (%) Infratentorial (%)	85.3 14.7	76.5 23.5	1.8 (0.7–4.7); 0.289
**Stroke diagnosis**			
PACI (%) TACI (%) POCI (%) LACI (%)	44.2 3.3 14.2 38.3	41.7 5.5 0 52.8	1.1 (0.5–2.4); 0.850 0.6 (0.1–3.3); 0.622 12.3 (0.7–210.6); 0.013 0.6 (0.3–1.2); 0.130
**Volume of stroke lesion (cc) (mean ± SD)**	6.9 ± 16.2	9.1 ± 27.3	0.367
**Institutionalization on discharge (%)**	11.3	25.0	2.6 (1.1–6.3); 0.046
**Barthel Index on discharge (median (IQ range))**	90 (90–100)	90 (80–100)	0.046
**Barthel Index at follow-up visit (median (IQ range))** ≥90 points (%)	100 (90–100) 82.8	100 (80–100) 65.2	0.004 9.0 (3.4–24.0); <0.001
**MNA-sf at follow-up visit (median (IQ range))**	13 (12–14)	12 (11–13)	0.033
**EQ-5D-3L:**			
Mobility dysfunctions (%)	14.2	30.9	2.7 (1.2–6.2); 0.021
Self-care dependency (%)	13.3	28.6	2.6 (1.1–6.1); 0.033
Dependency in daily life activities (%)	14.2	26.2	0.5 (0.2–1.1); 0.096
Pain/discomfort (%)	33.3	54.8	2.4 (1.2–5.0); 0.017
Anxiety/depression symptoms (%)	46.7	62.0	0.5 (0.3–1.1); 0.108
Perception of healthy status (mean ± SD)	72.8 ± 16.8	60.0 ± 21.0	< 0.001

NIHSS: National Institute of Health Stroke Scale; MCA: Middle cerebral artery; PACI: Partial anterior circulation infarct; LACI: Lacunar infarct; POCI: Posterior circulation infarct; TACI: Total anterior circulation infarct; EQ-5D-3L: European Quality of Life Scale; IQ: Interquartile.
